# Developmental Testicular Expression, Cloning, and Characterization of Rat HDAC6 In Silico

**DOI:** 10.1155/2017/5170680

**Published:** 2017-10-19

**Authors:** Pratibha Verma, Omshree Shetty, Sweta Parab, Karen Menezes, Priyanka Parte

**Affiliations:** Department of Gamete Immunobiology, ICMR, National Institute for Research in Reproductive Health, Mumbai 400012, India

## Abstract

We had previously reported presence of histone deacetylase 6 (HDAC6) in sperm and demonstrated its tubulin deacetylase activity and role in sperm motility in rat. In the present study we report its abundant expression in testis, epididymis, accessory sex organs, brain, and adrenal. In the testis, HDAC6 transcript and protein were observed throughout development. We therefore cloned the gene from rat testis using primers for* hdac6* (accession number XM_228753.8) in order to determine the role of acetylation/deacetylation in spermatogenesis. The cloned rat* hdac6* gene is ~3.5 kb with 28 exons and 1152 amino acids. We noted 4 single nucleotide polymorphisms (SNPs) on exons 2 (G/A), 5 (A/G), 7 (T/C), and 26 (G/T), respectively, in this sequence when compared to XM_228753.8. These were further validated at both cDNA and gene level. These SNPs resulted in 2 amino acids changes, namely, glycine → arginine and valine → phenylalanine at protein level. Cloned* hdac6* overexpressed in HEK293T cells demonstrated significant overexpression by IIF. Alpha-tubulin acetylation analysis of the overexpressed cell lysate demonstrated that the protein was bioactive. This is the first study showing the ontogenic expression in the testis and reporting experimentally validated sequence of rat HDAC6 and its structural and functional annotation in silico. This sequence has been submitted to GenBank (Accession number Rattus KY009929.1).

## 1. Introduction

Acetylation and deacetylation are crucial protein modifications which are increasingly gaining importance due to their contributions to many cellular processes. These modifications are maintained by enzymes acetyl transferases and deacetylases, respectively. Histone deacetylase 6 (HDAC6) is a class IIb HDAC protein identified to be predominantly cytoplasmic. Many cytoplasmic proteins such as *α*-tubulin, HSP90, and cortactin have been identified as its substrates [1–3]. It is distinct from other HDACs in that it has two catalytic domains arising due to duplication of class I/II homology domain and Zinc finger ubiquitin binding domain at C terminus. HDAC6 has been shown to be abundantly expressed in the testis and brain in mice [4]. The human ortholog expressed as a 131-kD protein does not coimmunoprecipitate with other HDACs or transcription factors [5]. It is 92% identical in rat and mouse and 77% identical in human and rat. Human HDAC6 has 1215 amino acid, while that of mice has 1149 amino acid. The SE14 tetradecapeptide repeating domain (SE14) of human HDAC6 is responsible for its intracellular retention and tau interaction which is absent in mice and rat [6, 7]. This gene is expressed 5.3 times and 2.6 times more than the average gene in human and in mouse, respectively.

While human and mouse* hdac6* have been well characterized and annotated due to extensive literature and experimental evidences available, there is no information about rat* hdac6*, due to unavailability of cDNA clone [6]. According to NCBI, Aceview (https://www.ncbi.nlm.nih.gov/IEB/Research/Acembly AceView: integrative annotation of cDNA-supported genes in human, mouse, rat, worm, and* Arabidopsis*), there are 574 cDNA clones isolated from various tissues substantiating the 33 variants of human gene HDAC6 and 702 GenBank accessions, while for mouse 270 cDNA clones from various organs and 289 GenBank accessions have been reported.

HDAC6 and alpha-tubulin acetyl transferase 1 (*α*TAT1) have been identified as *α*-tubulin deacetylase and *α*-tubulin acetyltransferase, respectively [1, 8]. Our group has reported, for the first time, the presence of HDAC6 in sperm and demonstrated its tubulin specific deacetylase activity and its role in sperm motility in Holtzman rat [9]. Not much is known about the role of HDAC6 during different stages of spermatogenesis. It is noteworthy that HDAC6 knock-out (HDAC6 KO) mice demonstrate no adverse effect on spermatogenesis despite HDAC6 being abundant in normal testis. The HDAC6 KO mice have been shown to have hyperacetylated tubulin in most tissues but are viable and normal indicating a negligible role for HDAC6 in normal development [10]. On the other hand, tubulin acetyltransferase *α*tat1 KO mice, although viable and developing normally, show significantly reduced sperm motility and fertility [8]. The status of HDAC6 in these mice is not known. Our data from individuals with poor sperm motility also show significantly reduced acetylation of alpha-tubulin in these individuals [11]. Our own observations as well as observations by others show an association of hypoacetylation of *α*-tubulin with poor sperm motility [12]. Recent report by Yu et al. further endorse the significance of tubulin acetylation to sperm motility, as the acetylation levels differed between normozoospermic and asthenozoospermic individuals [13]. Taking cues from our own observations and published reports, we believe that while hyperacetylation as demonstrated by the HDAC6KO mouse model may not affect spermatogenesis, HDAC6 overexpression-induced hypoacetylation may likely affect spermatogenesis. In order to understand the role of acetylation/deacetylation in spermatogenesis and sperm motility we have cloned, overexpressed rat HDAC6 and demonstrated that the overexpressed protein is catalytically active. We first studied the expression profile of rat HDAC6 in various tissues and the presence of HDAC6 protein and transcript in the testis. By cloning and experimental validation by sequencing we identified 4 nucleotide differences at cDNA level when compared with the sequence available in NCBI (XM_228753.8). We further validated these SNPs at gene level and further by Sanger sequencing. Structural and functional annotation was done bioinformatically.

## 2. Materials and Methods

### 2.1. Animals

Neonatal, prepubertal, pubertal, and adult male Holtzman rats were used. Food and water were provided ad libitum. Rats were housed in groups of four/cage under conditions of 12 h light and 12 h dark. The study was approved by the Institutional Animal Ethics Committee (IAEC).

### 2.2. Expression Profile of HDAC6 Protein and Transcript

For studying the expression profile of HDAC6 in rat tissues, testis, epididymis, seminal vesicles, prostrate, brain, adrenal, heart, lung, liver, kidney, and stomach were neatly dissected from adult Holtzman rats and snap frozen in liquid nitrogen. Following this, the tissue lysates was prepared in HDAC6 lysis buffer as described above, and 40 *μ*g protein lysates was resolved by electrophoresis on 10% sodium dodecyl sulfate- (SDS-) polyacrylamide gels and blotted to nitrocellulose membranes. These blots were further probed with HDAC6 antibody raised in-house and also using a commercial antibody (Sigma, Missouri, USA) and as described previously [9]. The same protocol was followed for determining the ontogenic expression of the protein on days 0 (at birth), 10, 20, 30, 45, 60, and 90, in rat testis.

Reverse transcription-PCR (RT-PCR) was employed to investigate the ontogenic expression of hdac6 transcript on days 0 (at birth), 10, 20, 30, 45, 60, and 90, in rat testis. One *μ*g of the respective total RNA was reverse transcribed to cDNA and 250 ng of this cDNA was amplified by PCR using CAGCTAACCAGACCACGTCA and TAGTAGGCCCTCCTCGGATT as the forward and reverse primers, respectively, at annealing temperature of 59°C for 1 min. “Reagent” control as well as “No RT” control was incorporated to check for any contamination from reagents used, or any hdac6 amplification from genomic DNA.

### 2.3. Extraction of RNA and cDNA Synthesis

For cloning, 2.5–3-month adult Holtzman rat testes were used to extract the RNA. Reverse transcription-PCR was employed to generate the* hdac6* coding region, using cDNA synthesized from RNA using Advantage cDNA synthesis kit (Takara bio, Mountain view, CA, USA). RNA was extracted using TRIzol (Invitrogen, California, USA) as per manufacturer's protocol and eluted using autoclaved DEPC treated water. The purity and concentration of the RNA was determined spectrophotometrically at 260 and 280 nm. For cDNA synthesis, 1 *μ*g of RNA was reverse transcribed using reverse transcriptase and oligo dT primers at 42°C for 1 h followed by denaturation at 94°C for 5 min and then snap chilled on ice. In order to rule out genomic DNA contamination a “no reverse transcriptase” (No RT) control was also included during the cDNA synthesis. PCR primers for* hdac6* were designed based on GenBank accession number XM_228753.8. Restriction enzyme site sequences encoding EcoR1 and Spe1 were included in forward and reverse primer, respectively. The forward and reverse primers used for* Hdac6* were TAGTTACATA*GAATTCCC ***ATG**ACCTCCACCGGCCAA and TTAAATGCG*ACTAGT*TCA**TTA**CTGCAAGTGCGGCATGCCCT wherein restriction sites are represented in italics and start and stop codons in bold.

### 2.4. PCR for Full Length* hdac6* ORF

The PCR for full length HDAC6 ORF was done using Phusion enzyme (New England Biolabs, Massachusetts, USA) under the following conditions: initial denaturation at 98°C for 45 sec, followed by denaturation at 98°C for 10 sec, annealing at 53.1°C for 30 sec, and extension at 72°C for 2.5 min for 35 cycles followed by final extension at 72°C for 10 min. The PCR product was electrophoresed on 0.8% agarose gel. This band was excised from the gel and purified using Nucleospin gel extraction kit as per the manufacturers protocol (Takara Bio, California, USA).

### 2.5. Cloning into pJET Cloning Vector & Sequencing

pJET cloning kit (Fermentas, Massachusetts, USA) was used for cloning of* hdac6* ORF. Briefly, the 3.5 Kb band excised and extracted from the gel was cloned into pJET cloning vector in 1 : 3 ratio and the mixture incubated at 22°C for 1 h. Ligation mixture was ethanol precipitated and then transformed in Top10 competent cells and the cells plated on LB agar containing 100 ug/ml ampicillin and incubated at 37°C overnight. Positive colonies were inoculated in 2 ml L.B broth containing 100 ug/ml ampicillin and incubated at 37°C, 230 rpm O/N. Plasmids containing the gene of interest were extracted by alkaline lysis. Positive clones were confirmed by restriction digestion of the isolated plasmid with EcoRI HF and SpeI-HF (New England Biolabs, Massachusetts, USA) sequentially, at 37°C overnight and electrophoresing the digested products on 0.8% agarose gels. Overlapping primers were designed for* hdac6* ORF using Primer 3 software to amplify and sequence the 3.56 kb gene by Sanger sequencing (Table 1).

### 2.6. PCR Amplification of Exons

Primers were designed for specific exons 3, 5, 7, and 26 using Primer3 software (Table 2). PCR amplification was done using 2x master mix (Kappa, Massachusetts, USA). Towards this, genomic DNA was isolated from rat tails by Phenol-Chloroform isolation (Hi Media, Mumbai, India) followed by isopropanol precipitation and* Hdac6* DNA was amplified using primers to the specific exons. The amplified products were resolved on 1.2% agarose gel. PCR amplified DNA was cleaned using PCR clean-up kit following the kit protocol (Promega, Wisconsin, USA), sequenced, and verified using nucleotide BLAST tool.

### 2.7. Subcloning into Expression Vector

The* Hdac6* ORF was subcloned into expression vector pLVX-IRES-zSGREEN having Ef1*α* as promoter (Takara bio, California, USA).* Hdac6* was ligated into pLVX vector in a 1 : 3 ratio using Long DNA ligation kit (Takara bio, California, USA). The ligation mixture was transformed into NEB stable competent cells (New England Biolabs, Massachusetts, USA), plated on LB agar plates containing ampicillin and incubated at 30°C overnight. The colonies obtained were screened by culturing them at 30°C, 230 rpm overnight, and isolating plasmid by alkaline lysis followed by digestion with EcoR1HF and Spe1HF and resolving on 0.5% gel. 1 kb plus ladder was used for determining size of the digested products (Fermentas, Massachusetts, USA). Sanger sequencing was done to confirm the sequence at gene level. The empty vector and pLVX vector containing* hdac6* gene were isolated and transfected into HEK293T cells. The cells were harvested 48 h after transfection.

### 2.8. Tissue Culture and Transfection

HEK293T cells were maintained at 37°C with 5% CO_2_ in DMEM supplemented with 10% FBS (Invitrogen, California, USA), 1% pen-strep, and nonessential minimal amino acids (Himedia, Mumbai, India). HEK293T cells were seeded a day prior to transfection, in 24 well plate. For transfection, plasmid was isolated using Qiagenmaxiprep Endotoxin free kit (Qiagen, Hilden, Germany). Cells were transfected with either hdac6 containing vector or empty vector using Xtreme gene reagent (Roche, Massachusetts, USA) at 37°C and 5% CO_2_ as per manufacturer's instructions. Forty-eight hours after transfection, cells were harvested by digesting them with 0.25% trypsin EDTA.

### 2.9. Western Blot Analysis

For western blot analysis, 48 h after transfections, cells were lysed in HDAC6 lysis buffer, pH 7.4 containing 15 mM Tris-HCl, 0.34 M sucrose, 60 mM KCl, 15 mM NaCl, 0.65 mM spermidine, 2 mM EDTA, 0.5 mM EGTA, 0.05% Triton X-100, 1 mM dithiothreitol [DTT], and 0.5 mM phenylmethylsulfonyl fluoride [PMSF] as described previously [9]. Protein quantification was done by Bradfords method [14]. 25 *μ*g of cell lysates was electrophoresed on 10% SDS-PAGE and transferred onto the nitrocellulose membrane. Blocking of nonspecific sites was done by incubating the blots in 5% nonfat dry milk (NFDM) at RT with gentle rocking for 1 h. Membranes were then incubated with monoclonal antibodies to Ac *α*-tubulin or *α*-tubulin (Sigma, Missouri, USA) at 1 : 5000 dilution in 0.1 M phosphate buffer saline (PBS) containing 0.1% Tween 20 and 1% nonfat dry milk (PBST-NFDM) overnight at 4°C. After washes in 0.1 M PBST, the blots were incubated with 1 : 3000 dilution HRP-conjugated rabbit anti-mouse antibody for 1 h at RT. The antigen antibody signals were detected by enhanced chemiluminescence (GE Healthcare, Illinois, USA).

### 2.10. Indirect Immunofluorescence

For indirect immunofluorescence detection of HDAC6 expression, the HEK293T cells were seeded on coverslip. The cells were transfected as described above and 48 h after transfection, cells were washed with 1x Dulbeco phosphate buffer saline without calcium and magnesium (DPBS; Invitrogen, CA, USA), fixed with 4% PFA for 10 min, permeabilized by using 0.1% Triton X-100, and probed with a 1 : 5 diluted rabbit polyclonal anti-rat HDAC6 antibody (Thermo Fisher Scientific, Ill., USA). Alexa 594 swine anti-rabbit antibody was used at 1 : 100 dilution as the secondary antibody and 4,6-diamidino-2-phenylindole (DAPI) was used to stain the cell nucleus. Images were taken using LSM 510 Meta Confocal microscope (Carl Zeiss, Oberkochen, Germany). Image J software was used for analysis of images. HDAC6 intensity of GFP positive cells was quantified for mock transfected and* hdac6*-transfected cells and the mean fluorescence intensity per cell (MFI) was calculated for both the groups. The significance of the difference in intensity was determined by Student's* t*-test.

### 2.11. In Silico Analysis of Rat HDAC6

Bioinformatic analysis of HDAC6 was done using the MUpro, COPid, and PredictProtein tools [15–17].

## 3. Results

### 3.1. HDAC6 Profiling in Tissues and Ontogenic Expression of the Transcript and Protein in the Testis

**Figure 1:**
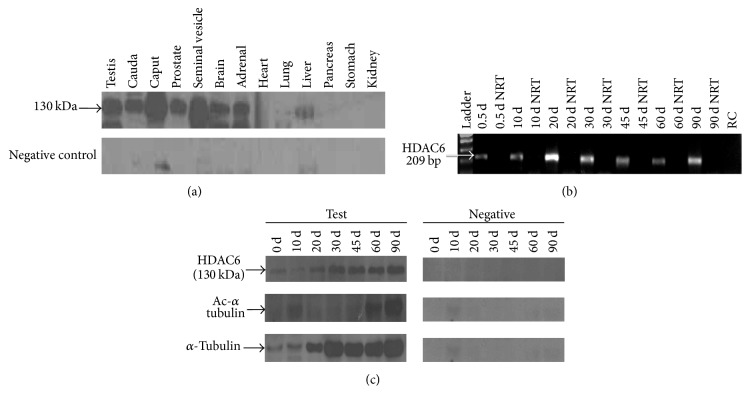
*Western blot analysis for HDAC6*. Western blot analysis was done to detect HDAC6 in multiple rat tissues and in testis from day 0 until day 90. The transcript for HDAC6 in the testis on these days was detected by RT-PCR. A band of 130 kDa was observed in testis, epididymis (cauda and caput region), prostrate, seminal vesicles, brain, adrenal, lungs, and liver (a). A 209 bp band indicative of the* hdac6* transcript is seen in the testis from birth till day 90 (b). The upper panel, middle panel, and lower panel show the 130 kDa band of HDAC6, 55 kDa band for acetylated alpha-tubulin (Ac *α*-tubulin), and alpha-tubulin (*α*-tubulin), respectively, from birth to 90 d (c). “Reagent” control (RC) as well as “No RT” (NRT) control were incorporated to account for nonspecific amplification, if any.

We determined the presence of HDAC6 in multiple rat tissues, namely, testis, epididymis, prostrate, seminal vesicles, brain, adrenal, heart, lungs, liver, pancreas, stomach, and kidney, respectively, using the rabbit polyclonal HDAC6 antibody raised in-house. HDAC6 was abundantly present in testis, epididymis, prostrate, seminal vesicles, brain, and adrenal. Weak expression was seen in heart, lungs, and liver (Figure 1(a)). With the commercial antibody, the protein band was noted only in testis, epididymis, brain, and adrenal gland (data not shown).

RT-PCR to investigate the expression of the transcript for HDAC6 in 0.5, 10, 20, 30, 45, 60, and 90 d rat testis indicates an expression of the transcript from d 0.5 postnatal to adulthood. “Reagent” control (RC) as well as “No RT” (NRT) control showed no contamination from reagents used, or any HDAC6 amplification from genomic DNA. A transcript with a product size of 209 bp was observed in all the tissues studied. Western blot analysis of the protein indicates an increased expression of protein from days 0 to 90 d whereas expression of acetylated alpha-tubulin was observed only in d60 and d90 rat testis (Figures 1(b) and 1(c)).

### 3.2. Cloning of Rat* hdac6* ORF

**Figure 2:**
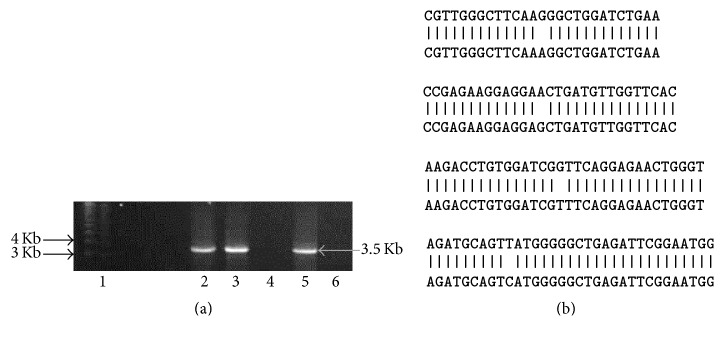
*Amplification of hdac6 cDNA from rat testis*. RNA was isolated from testis of 28-day-old adult Holtzman rat, reverse transcribed, and the cDNA amplified by PCR using primers designed for accession number XM_228753.8. (a). “Reagent” control (RC) as well as “no reverse transcriptase” (No RT) control were incorporated to account for nonspecific amplification. Lane 1: 1 Kb plus ladder; Lanes 2, 3, and 5: amplified* Hdac6*; Lane 4: No RT control; Lane 6: reagent control. Rat* Hdac6* cDNA is ~3.5 kb. Gel extracted PCR product (b). The sequences obtained were aligned using BLAST tool. Four nucleotide differences were observed in different regions of the nucleotide sequences with respect to XM_228753.8.

**Figure 3:**
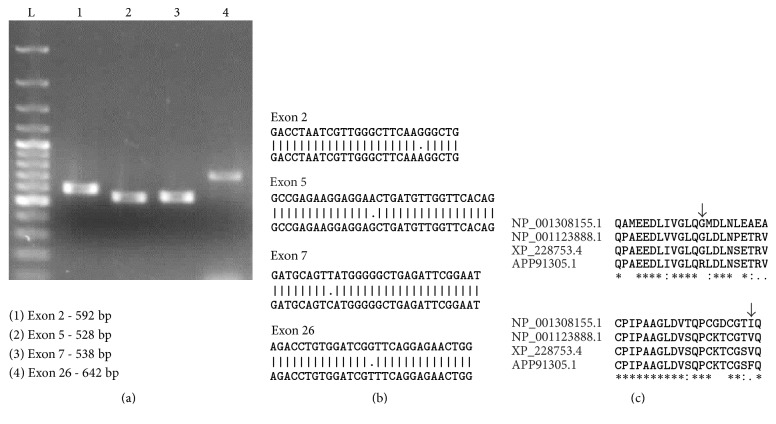
*Amplification, sequencing and analysis of exons 2, 5, 7, and 26*. Exons 2, 5, 7, and 26 were amplified by PCR using primers specific to these regions (a). L-100 bp ladder. PCR products were sequenced by Sangers method. All the 4 nucleotide differences were also present at genomic DNA level. Representative image of exon specific amplification (b). Alignment of exon specific sequencing results with human, mouse, and rat (predicted) sequence shows 2 amino acid residue changes (c).

RNA was extracted from adult Holtzman rat from testis, reverse transcribed into cDNA, and amplified by PCR. A 3.56 kb band was obtained (Figure 2(a)). We cloned* hdac6* using testicular RNA into pJET cloning vector and transformed in TOP10 competent cells. Plasmids were isolated from the positive colonies and double digested with EcoRI HF and SpeI-HF. Two bands of 2.9 Kb and 3.5 kb were observed for vector and* hdac6* gene, respectively (data not shown). The full length 3.56 kb gene was sequenced by Sanger sequencing and the sequences were confirmed by pairwise alignment using nucleotide BLAST with XM_228753.8 (data not shown). We noted four nucleotide differences between accession number XM_228753.8 and the sequencing results obtained by us from Holtzman rat (Figure 2(b)). Five colonies were sequenced and these differences were noted in all the colonies (data not shown). To check and further validate that these sequence differences are indeed present at gene level, we amplified exons 2, 5, 7, and 26. The PCR products were sequenced and the sequences were aligned using nucleotide BLAST tool (Figures 3(a) and 3(b)). 10 random rats were screened, and all the four nucleotide level differences present at cDNA level (G/A, A/G, T/C, and G/T) were also found to be present in the gene level at the respective exons. Aligning of XP_228753.8 with that obtained by us for the Holtzman rat showed 2 amino acid changes, namely, glycine to arginine and valine to phenylalanine in the Holtzman rat (Figure 3(c)).

### 3.3. Subcloning and Detection of Overexpression and Deacetylase Activity of HDAC6

**Figure 4:**
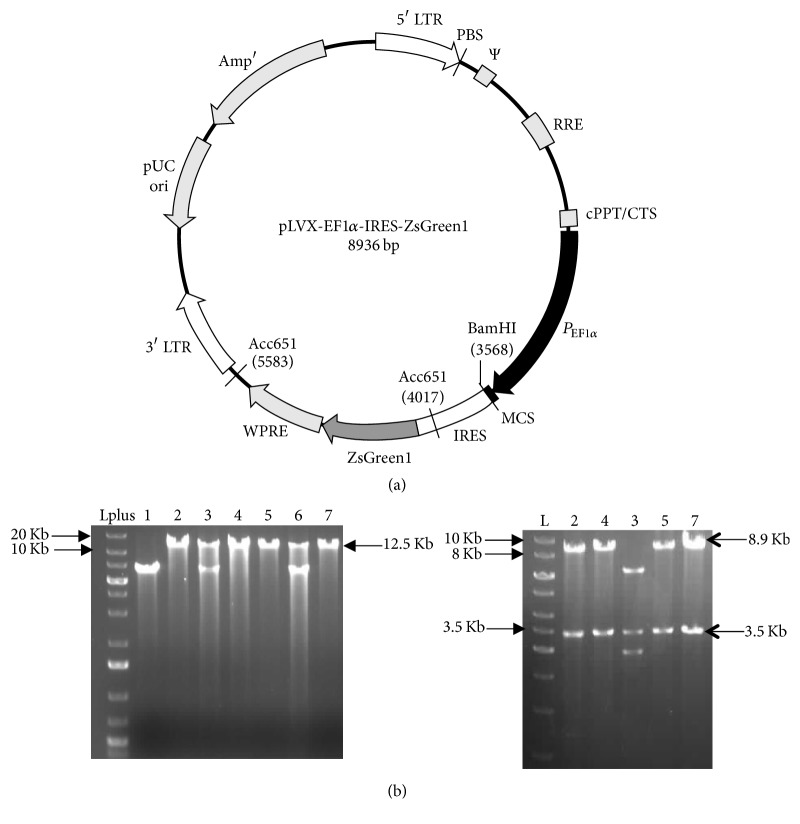
*Subcloning of rat hdac6 gene in pLVX-EF1α-IRES-Zsgreen vector*. Vector map of pLVX-EF1*α*-IRES-Zsgreen expression vector (a). The* hdac6* ORF was cloned into the expression vector and transformed into NEB stable competent cells. Plasmids were isolated from the colonies and digested with EcoRI. Representative figure showing some of the colonies 1–7 (b-left panel). The positive colonies (numbers 2, 4, 3, 5, and 7) were double digested with Spe1HF and EcoRI HF (b-right panel). (b) shows the bands for 8.9 Kb vector and 3.5 Kb* hdac6* from representative colonies. Lplus: 1 Kb plus ladder; L: 1 Kb ladder.

**Figure 5:**
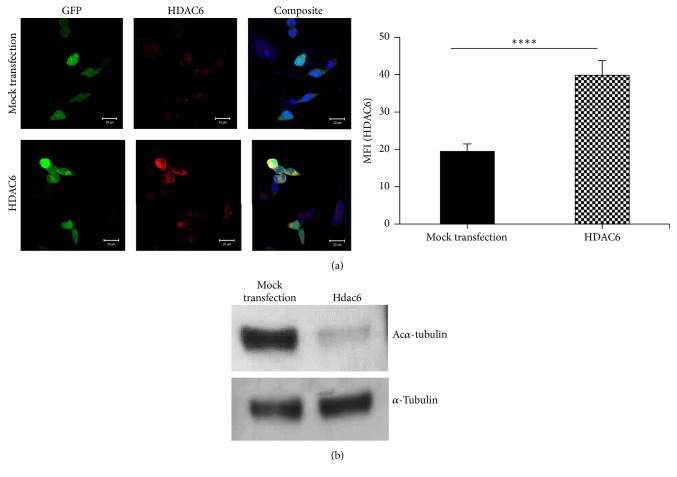
*HDAC6 protein overexpression and its effect on tubulin acetylation in HEK 293T cells*. HEK293T cells were transfected with either the empty vector (mock transfection) or pLVX-EF1*α*-hdac6–IRES-zSGREEN and then processed for either indirect immunofluorescence detection of HDAC6 expression or western blot analysis. A significantly increased expression of HDAC6 was observed in the HDAC6-transfected cells (a-left panel). Quantification of HDAC6 expression was done by Image J analysis. Graphical presentation of the same is seen (a-right panel). Values are mean ± SEM. ^*∗∗∗∗*^*P* < 0.00005. The transfected cell lysates were electrophoresed on 10% SDS-PAGE and transblotted for detection of acetyl *α*-tubulin and *α*-tubulin using their respective antibodies (b). The blot is a representative image of 3 independent experiments.

We studied the activity of the HDAC6 protein, by subcloning the gene into pLVX-IRES-zSGREEN vector having Ef1*α* as promoter (Figure 4(a)). Colonies were screened by digestion of the isolated plasmid with EcoR1HF. The band at ~12.5 kb suggests that ligation was successful. This was confirmed by sequential double digestion with EcoR1 followed by Spe1 wherein two bands of 8.9 kb and 3.5 kb were observed (Figure 4(b)). The plasmid containing the gene was transfected into HEK293T cells. Indirect immunofluorescence detection of HDAC6 expression in these cells demonstrated significantly higher expression of HDAC6 in the cells transfected with* hdac6* as compared to mock transfected cells (*P* < 0.0001; Figure 5(a)). Western blot analysis for the acetylated alpha-tubulin levels in these cells demonstrated a reduction in acetyl *α*-tubulin expression in lysates of HDAC6 overexpressing HEK293T cells compared to cells transfected with empty vector thus indicating that* hdac6* gene cloned by us is catalytically active at protein level (Figure 5(b)).

## 4. Discussion

The importance of acetylation in spermatogenesis and sperm motility has been very well documented [11–13]. Sperm flagella show high levels of tubulin acetylation, a phenomena which is conserved among different species from insect to mammals including humans [9, 11, 13, 18–20]. Despite high tubulin acetylation levels in the sperm flagella, we noted the presence of HDAC6, a tubulin deacetylase in the sperm flagella, and further provided evidences for its role in sperm movement [9]. In the present study, we determined the presence of HDAC6 in multiple rat tissues. HDAC6 was abundantly present in testis, epididymis, brain, and adrenal as seen with both the antibodies and additionally in the prostate and seminal vesicle with the antibody raised by us. Weak expression was seen in heart, lungs, and liver using the antibody raised by us but not with the commercial antibody. The abundant expression in the testis corroborated with observations of the* hdac6* transcript in mice previously reported [4]. We next sought to determine the ontogeny of this testicular expression right from birth until adulthood. The transcript as well as the protein expression increased from birth to 90 d concurrent with increase in alpha-tubulin. Interestingly although alpha-tubulin increased with abundant expression seen from day 30 onwards, copious expression of acetylated alpha-tubulin was observed only in d60 and d90 rat testis (Figures 1(b) and 1(c)). Previously, the presence of HDAC6 has been shown in spermatogonia of 6-day-old mice and in pachytene spermatids and round and elongating spermatids of adult mice [21]. The abundant expression of HDAC6 in testis indicates a crucial role for HDAC6 in spermatogenesis. Strangely, HDAC6 KO mice display a normal phenotype suggesting a redundant role for HDAC6 [10]. On the other hand, tubulin acetyltransferase *α*tat1 KO mice, although viable and developing normally, show significantly reduced sperm motility and fertility [8]. We have previously reported that acetyl *α* tubulin was significantly reduced in individuals with poor sperm motility [11]. In light of this knowledge, we hypothesized that while hyperacetylation as demonstrated by the HDAC6KO mouse model may not affect spermatogenesis, HDAC6 overexpression-induced hypoacetylation may likely affect spermatogenesis. We therefore overexpressed rat testicular HDAC6. RNA was extracted from adult Holtzman rat from testis, reverse transcribed into cDNA, and amplified by PCR. A 3.56 kb band was obtained (Figure 2(a)). This was cloned into pJET cloning vector and transformed in TOP10 competent cells, and the plasmids from the positive colonies were double digested with EcoRI HF and SpeI-HF. Two bands of 2.9 Kb and 3.5 kb were observed for vector and* hdac6* gene, respectively (data not shown).

The sequences of the full length 3.56 kb gene thus obtained were confirmed. Four nucleotide differences were noted between accession number XM_228753.8 and the sequencing results obtained by us from Holtzman rat in all the colonies that were sequenced and also at the gene level. The cDNA sequence using ExPASY tool translated into 1152 amino acids with 28 exons. The protein sequence available at NCBI (XP_228753.8 in REfseq) is the one predicted for Norway rat. When this was aligned with our sequence, 2 amino acid changes, namely, glycine to arginine and valine to phenylalanine, were observed in the Holtzman rat sequence. These two 2 SNPs were seen in exon 2 (G/A) and exon 26 (G/T), respectively, both being missense mutation, resulting in amino acid change. SNPs present in exons 5 (A/G) and 7 (T/C) are synonymus mutations (Figure 3(c)). On aligning the sequence for Holtzman rat with that of human, mice, and Norway rat, we noted that glycine was conserved across species though not in Holtzman rat. Also Holtzman rat had an aromatic amino acid residue, namely, phenylalanine in place of the aliphatic amino acid residues isoleucine and valine (Figure 3(c)). We therefore determined whether or not the cloned gene was active at protein level. Since CMV promoter is prone to silencing* in vivo/in vitro* [22–24], we cloned* hdac6* under Ef1*α* promoter into pLVX-IRES-ZsGreen expression vector. A significantly higher expression of HDAC6 in the cells transfected with* hdac6* as compared to mock transfected cells confirmed the overexpression (*P* < 0.0001; Figure 5(a)). Acetyl *α* tubulin in these cells was significantly reduced compared to cells transfected with empty vector thus indicating that* hdac6* gene cloned by us is catalytically active at protein level (Figure 5(b)).

**Figure 6:**
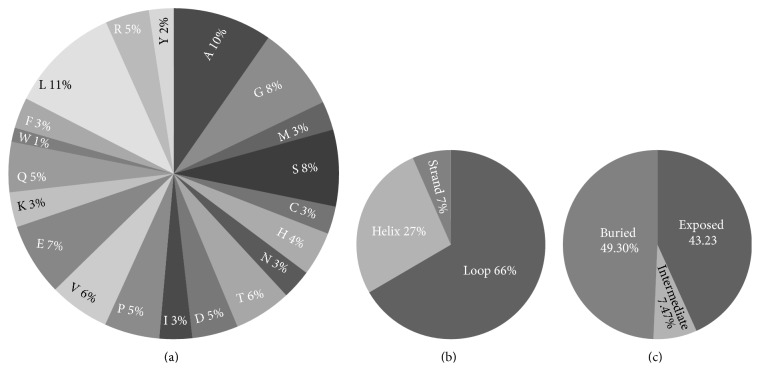
*Structure annotation of HDAC6 using ProteinPredict tool*. The sequence obtained by us for the Holtzman rat was submitted for analysis of amino acids, secondary structure, and solvent accessibility of the residues using the ProteinPredict tool. The values obtained were plotted and represented as a pie chart; percentage contribution of amino acid in the hdac6 sequence (a), percentage of secondary structure elements (b), and percentage solvent accessibility of amino acids (c).

Using MUpro tool, we deduced that both amino acid changes increased the stability of the protein. Further analysis of this sequence using PredictProtein tool indicated that there are no disulfide bonds in the sequence despite the presence of 31 cysteine residues. The percentage of each amino acid present in the sequence, percentage secondary structure, elements, that is, helix (includes alpha-, pi-, and 3_10_-helix), (beta-) strand (extended strand in beta-sheet conformation), and loop and solvent accessibility of the residues, was also determined (Figures 6(a)–6(c)). Gene ontology analysis indicated HDAC6 involvement in biological process such as cellular macromolecule metabolic process, primary metabolic process, heterocycle metabolic process, and regulation of nucleobase-containing compound metabolic process. Molecular functions were histone deacetylase activity, hydrolase activity, acting on carbon-nitrogen (but not peptide) bonds, protein deacetylase activity, protein binding, and hydrolase activity. Organelle involves intracellular organelle, membrane-bounded organelle, nucleus, and intracellular cytoplasm.

HDAC6 sequence has various posttranslational modification sites which have been summarized in Table 3. As mentioned earlier HDAC6 has two catalytic domains. Driven by the quest to know the sequence level conservation of both the catalytic domains, multiple sequence alignment specifically of both the catalytic domains was done with mouse and human hdac6 sequence. It was observed that the rat CD1 lacked the terminal 49 amino acids. Likewise in CD2, it has additional 49 amino acids at the start of CD2 and lacked the 49 amino acids at the end of CD2 (data not shown).

**Figure 7:**
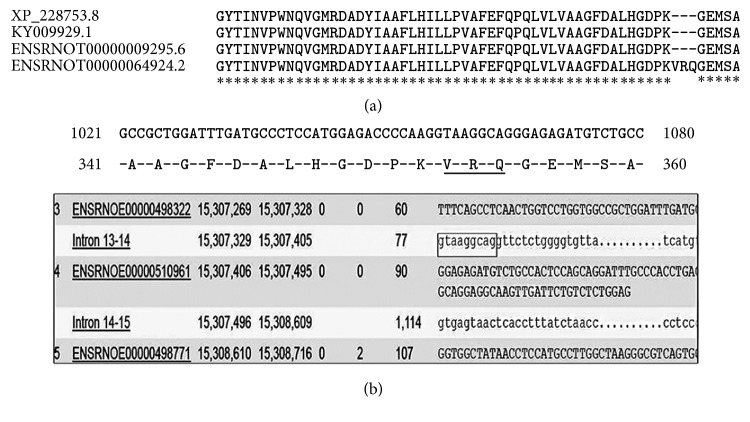
*Comparison of sequences from Ensemble database*. The sequence obtained for Holtzman rat (Rattus KY009929.1), sequences from the Ensemble databases, namely, ENSRNOT00000009295.6 and ENSRNOT00000064924.2 and XM_228753.8, were aligned. Three amino acids valine, arginine, and glutamine are seen in ENSRNOT00000064924.2 (a). The region on ENSRNOT00000064924.2 showing the nucleotides** GTAAGGCAG** corresponding to amino acids V, R, and Q which is seen in exon 12 (b-upper panel). A snapshot of the intronic region of Hdac6 transcript ENSRNOT00000009295.6 shows that these 3 amino acids lie in the intron 13-14 region (b-lower panel).

This is the first study reporting the ontogenic expression of HDAC6 protein and transcript in the testis. This study also provides novel information on the experimentally validated sequence of rat HDAC6. This sequence has been submitted to GenBank and the accession number provided by GenBank is Rattus KY009929.1. We have also identified 4 nucleotide differences as compared to the* hdac6* sequence in the Norway rat (XM_228753.8) available at NCBI. These differences are present in exon 2 (G/A), exon 5 (A/G), exon 7 (T/C), and exon 26 (G/T) in cDNA sequences, as well as in the gene. We have analyzed structural and functional annotation for rat* hdac6*, bioinformatically. The Ensemble database reports two HDAC6 sequences for rat, one comprising 1152 amino acids (ENSRNOT00000009295.6; 29 exons) and the other 1155 amino acids (ENSRNOT00000064924.2; 28 exons). In case of ENSRNOT00000009295.6, exon 1 represents the UTR region and coding starts from exon 2. On aligning these two protein sequences, we observed that ENSRNOT00000064924.2 showed three amino acids, valine (V), arginine (R), and glutamine (Q). It is perplexing that these 3 amino acids lie in the intronic region between exons 13 and 14 for ENSRNOT00000009295.6 but in the exon 12 for ENSRNOT00000064924.2 (Figure 7). Although our sequence also had 28 exons, we did not observe this sequence in exon 12.

We had earlier reported the significance of HDAC6 in sperm motility opening a new avenue for HDAC6. We had identified its presence in sperm and shown that sperm of asthenozoospermic individuals has reduced acetylation. We now show its expression across rat testicular development. Cloning of rat HDAC6 and demonstrating that it is bioactive have opened up opportunities to further elucidate its role in spermatogenesis. Work is currently being pursued in that direction. Research on HDAC6 is being vigorously pursued for its role in neurodegenerative diseases such as Alzheimer's, Parkinson's, and Huntington's diseases as well as in cancers and use of HDAC6 specific inhibitors has been demonstrated to be beneficial in animal models [25, 26]. The availability of cloned rat hdac6 would also facilitate research advances in these areas.

## 5. Conclusion

The study demonstrates HDAC6 expression in the testis right from birth until adulthood. It also highlights strain specific difference in the sequence of hdac6 of Holtzman rat as compared to Norway rat, identifying 4 SNPs. This is the first study reporting experimentally validated sequence of rat HDAC6 and documenting its structural and functional annotation in silico. This sequence has been submitted to GenBank and the accession number provided by GenBank is Rattus KY009929.1.

## Figures and Tables

**Table 1:** Primers used for sequencing full length rat *hdac6* gene.

Fragment length	Primer sequence	Product size (bp)
pJET	CGACTCACTATAGGGAGAGCGGC AAGAACATCGATTTTCCATGGCAG	
176–828	GCCAACCAGCTGAAGAAGAC GCGGTGGATGGAGAAATAGA	653
648–1269	TCAGCGCAGTCTTATGGATG GATAGAAGTCTGGGCGCTTG	622
1522–1882	AAGGCCACTGGGAGGCCACT TGTGCGCAGGCAAAGGTGCT	360
1643–2321	GGGCCACAGAAAAGATGAAA ATAATACGGCCACCAGCAAG	679
2027–2597	AGCTGCTCGCCATGCCCAAG AGCCAGGGCTCCTGACTGGG	571
2566–3125	CGCTCCGACCTCCCCAGTCA AAGCCCAAGCTCGGGGGACT	560

**Table 2:** Primers for amplification of exons.

S. number	Primer sequence	Product size	*Tm*	Exon
(1)	CACCTTTGCTAGAGCCGTTC TTTGGGGACAATTTGGTGAT	592	60	Exon 2
(2)	ATGAATGCTAGCAGGGAGGA TAGCCATGACGCAGATTCAG	528	59	Exon 5
(3)	GGGGTATCTCCCGTATCCAT TTCAGGAATCCACCAACACA	539	59	Exon 7
(4)	ATGCCGTGACTCCACTATCC AAGGTAAACAGGCAGCATGG	642	60	Exon 26

**Table 3:** Putative posttranslational modifications on rat HDAC6.

S. number	Type of PTM	Pattern	Number of sites
(1)	N-glycosylation site	N[^∧^P][ST][^∧^P]	6
(2)	cAMP- and cGMP-dependent protein kinase phosphorylation site	[RK]{2}.[ST]	2
(3)	Protein kinase C phosphorylation site	[ST].[RK]	10
(4)	Casein kinase II phosphorylation site	[ST].{2}[DE]	11
(5)	Tyrosine kinase phosphorylation site	[RK].{2,3}[DE].{2,3}Y	1
(6)	N-myristoylation site	G[^∧^EDRKHPFYW].{2}[STAGCN][^∧^P]	16
(7)	Amidation site	G[RK][RK]	1
